# Temporal and spatial requirements of Smoothened in ventral midbrain neuronal development

**DOI:** 10.1186/1749-8104-8-8

**Published:** 2013-04-26

**Authors:** Mianzhi Tang, Sarah X Luo, Vivian Tang, Eric J Huang

**Affiliations:** 1 Department of Pathology, University of California San Francisco, San Francisco, CA 94143, USA; 2 Program in Neuroscience, University of California San Francisco, San Francisco, CA 94143, USA; 3 Pathology Service 113B, VA Medical Center, San Francisco, CA 94121, USA

## Abstract

**Background:**

Several studies have indicated that Sonic hedgehog (Shh) regulates the expansion of dopaminergic (DA) progenitors and the subsequent generation of mature DA neurons. This prevailing view has been based primarily on *in vitro* culture results, and the exact *in vivo* function of Shh signaling in the patterning and neurogenesis of the ventral midbrain (vMB) remains unclear.

**Methods:**

We characterized the transcriptional codes for the vMB progenitor domains, and correlated them with the expression patterns of Shh signaling effectors, including Shh, Smoothened, Patched, Gli1, Gli2 and Gli3.

**Results:**

While Shh and its downstream effectors showed robust expression in the neurogenic niche for DA progenitors at embryonic day (E)8 to E8.5, their expression shifted to the lateral domains from E9.5 to E12.5. Consistent with this dynamic change, conditional mutants with region-specific removal of the Shh receptor Smoothened in the vMB progenitors (*Shh-Cre;Smo*^*fl/fl*^) showed a transient reduction in DA progenitors and DA neurons at E10.5, but had more profound defects in neurons derived from the more lateral domains, including those in the red nucleus, oculomotor nucleus, and raphe nuclei. Conversely, constitutive activation of Smoothened signaling in vMB (*Shh-Cre;SmoM2*) showed transient expansion of the same progenitor population. To further characterize the nature of Shh-Smoothened signaling in vMB, we examined the BAT-GAL reporter and the expression of *Wnt1* in vMB, and found that the antagonistic effects of Shh and Wnt signaling critically regulate the development of DA progenitors and DA neurons.

**Conclusion:**

These results highlight previously unrecognized effects of Shh-Smoothened signaling in the region-specific neurogenesis within the vMB.

## Background

The mechanisms that govern the patterning of the neural tube and the subsequent generation of diverse neuronal subtypes have attracted intense attention. Because of its highly conserved structure, the developing spinal cord has provided an elegant model system to identify cell intrinsic and extrinsic cues that control the expansion of progenitors and differentiation of neurons [1]. Sonic hedgehog (Shh) is a potent morphogen that controls the development of spinal cord [1,2]. It is well established that temporal adaption to the graded Shh signals determines the progenitor and neuron identity in the ventral spinal cord [3-5]. Furthermore, the transcriptional network acting downstream of Shh provides important clues to the molecular logics that govern the diversity of ventral neural tube development [6].

In addition to the spinal cord, Shh has also been shown to regulate cell fate, expansion, and self-renewal of progenitors in the ventral forebrain, midbrain, and midbrain/hindbrain boundary (MHB) [7-10]. For instance, exogenous Shh, together with fibroblast growth factor (FGF) 8, can induce midbrain dopaminergic (DA) neurons in culture [11,12]. Furthermore, fate-mapping studies show that Shh-expressing progenitors give rise to different neurons in the ventral midbrain (vMB) [13-16]. Indeed, several conditional mutants have been developed to remove Shh or the Shh receptor Smoothened using the *Engrail1-Cre* (*En1-Cre;Smo*^*fl/fl*^) mutation, which specifically targets the mid/hindbrain region [8,17]. These mutants show severe defects in the DA neurons, but it remains unclear if these defects are directly due to the effects of Shh in promoting DA neuron development or are caused by the loss of *FGF8* and by profound MHB patterning defects in *En1-Cre;Smo*^*fl/fl*^ mutants [18-20]. Thus, the exact role of Shh signaling in the development of DA and other progenitors in vMB remains unclear.

In this study, we used a set of transcription factors to define four distinct progenitor domains in vMB. Shh and its downstream effectors also showed robust expression in the neurogenic niche for DA progenitors at embryonic day (E)8 to E8.5, but their expression became progressively restricted to the lateral domains in vMB from E9.5 to E12.5. Interestingly, conditional mutants with vMB-specific removal of the Shh receptor Smoothened (*Shh-Cre;Smo*^*fl/fl*^) showed a transient reduction in DA progenitors and DA neurons at E10.5, but had more profound defects in neurons derived from the more lateral progenitor domains. Conversely, constitutive activation of Smoothened signaling in vMB (*Shh-Cre;SmoM2*) showed a transient expansion of the same progenitor population. The transient effects of Shh-Smoothened signaling in vMB were due to the antagonistic effects of Shh and Wnt signaling that critically regulate the development of DA progenitors and DA neurons. Together, our results provide comprehensive views of the effects of Shh signaling on neurogenesis in vMB.

## Methods

### Animals

All procedures were approved by the University of California, San Francisco Institutional Animal Care and Use Committee. *Shh-Cre*, *Smoothened*^*fl/fl*^ (Smo^*fl/fl*^), *SmoothenedM2* (*SmoM2*), *Rosa26* (*R26R*) and *BAT-GAL* mice (stock numbers 005622, 004526, 005130, 003474 and 005317, respectively; the Jackson Laboratory, Bar Harbor, ME, USA). To generate conditional mutant mice that lacked Smoothened in the ventral neurogenic niche for DA neurons, Smo^*fl/fl*^ mice were first crossed with *Shh-Cre* to generate *Shh-Cre;Smo*^*fl/+*^ mice, then *Shh-Cre;Smo*^*fl/+*^ mice were crossed with *Smo*^*fl/fl*^ to generate the *Shh-Cre;Smo*^*fl/fl*^ mutant. We also used the same Cre line to generate conditional mutants in which the constitutive active Smoothened receptor was expressed in the *Shh-Cre* domain (*Shh-Cre;SmoM2*).

### Histology and immunohistochemistry

Histology and immunohistochemistry (IHC) were performed as described previously with minor modifications [21,22]. Specifically, mouse embryos were collected E8.5, E9.5, E10.5, E11.5, and E12.5, then fixed in 4% paraformaldehyde (PFA) for 0.5 to 2 hours, followed by cryoprotection in 15 to 30% sucrose solutions, and sectioned on a cryostat (Leica, Heerbrugg, Switzerland). Primary antibodies in this study were: anti-Brn3a antibody (1:1,000; [23]), anti-β-galactosidase (β-Gal; 1:20; #40-1a; Developmental Hybridoma Study Bank (DHSB), Iowa City, IA, USA), anti-Foxa2 (1:500; #3143; Cell Signaling Technology, Danvers, MA, USA), anti-5-hydroxytryptamine (anti-5-HT; 1:500; #20080; ImmunoStar Inc., Hudson, WI, USA), anti-Lmx1a (1:1,000; gift of Dr Mike German, UCSF), anti-Islet1 (1:50; 39.4D5; DHSB), anti-Nkx2.2 (1:50; 74.5A5; DHSB), anti-Nkx6.1 (1:50; F55A10; DHSB), anti-Nurr1 (1:500; sc-990; Santa Cruz Biotechnology, Santa Cruz, CA, USA), anti-Olig2 (1:1,000; Gift of Dr David Rowitch, UCSF), anti-Pax6 (1:50; Pax6; DHSB), anti-Shh (1:200; #2207; Cell Signaling Technology), anti-Sox2 (1:200; AB5603; Millipore Corp., Billerica, MA, USA), and anti-tyrosine hydroxylase (anti-TH; 1:500; ab113; Abcam, Cambridge, MA, USA). For immunofluorescence staining, sections were incubated with primary antibody overnight, followed by secondary antibodies conjugated with Alexa fluorophores 488 and 568 (Invitrogen Corp., Carlsbad, CA, USA) for 1 hour to detect signals. For chromogen staining, sections were incubated with primary antibody overnight, followed by incubation for 1 hour with biotinylated IgG and avidin–biotin complex (Vector Laboratories, Burlingame, CA, USA). Diaminobenzidine (DAB) solution was used to visualize the results. Images were captured using a confocal microscope (LSM 510l Carl Zeiss 510 Microimaging, Jena, Germany), or a microscope (BX41 Olympus, Tokyo, Japan) equipped with a charge-coupled device (CCD) camera (DP70; Olympus).

### *In situ* hybridization

RNA probes for *in situ* hybridization were prepared using plasmids that contained cDNA for *Smoothened*, *Patched*, *Gli1*, *Gli2*, *Gli3* (gifts from Dr. Arturo Alvarez-Buylla, UCSF), *FGF8*, or *Wnt1*. The plasmids were linearized with appropriate restriction enzymes, and transcribed with SP6, T7, or T3 polymerase using digoxigenin (DIG)-labeling reagents and a DIG RNA labeling kit (Roche Diagnostics, Basel, Switzerland). For *in situ* hybridization, embryos were fixed overnight at room temperature in 4% PFA in diethylpyrocarbonate (DEPC)-treated PBS, cryoprotected in 15% and 30% sucrose, and embedded in optimal cutting temperature (OCT) compound, then sections were cut at 10 μm on Leica CM1950 crystat (Leica Microsystems, Buffalo Grove, IL, USA). During hybridization, sections were first post-fixed with 4% PFA, then washed with acetylation solution and 1% Triton X-100. Sections were incubated with hybridization buffer (Amresco LLC, Solon, OH, USA) for 2 to 4 hrs before applying hybridization buffer containing DIG-labeled riboprobes (200 to 400 ng/ml) at 65°C overnight. On the second day, slides were washed twice for 30 minutes each with 0.2 × SSC (0.1% Tween 20, pH 4.5) at 65°C, then twice for 10 minutes each with a solution of 100 mmol/l Maleic acid, 150 mmol/l NaCl, 2 mmol/l levamisole and 0.1% Tween (pH 7.5). Sections were blocked for 1 hour and incubated with anti-DIG antibody overnight at 4°C. For visualizing the *in situ* hybridization results, we used BM purple (Boehringer Mannheim, Mannheim, Germany). Finally, the slides were dried at room temperature and mounted (Clear Mount; Electron Microscopy Sciences, Hatfield, PA, USA).

### Statistical analyses

Data were analyzed by two-tailed Student’s *t* test. Values were expressed as mean ± s.e.m. Changes were considered as significant at *P*<0.05.

## Results

### Transcriptional codes define distinct temporal and spatial progenitor domains in the early embryonic ventral midbrain

**Figure 1:**
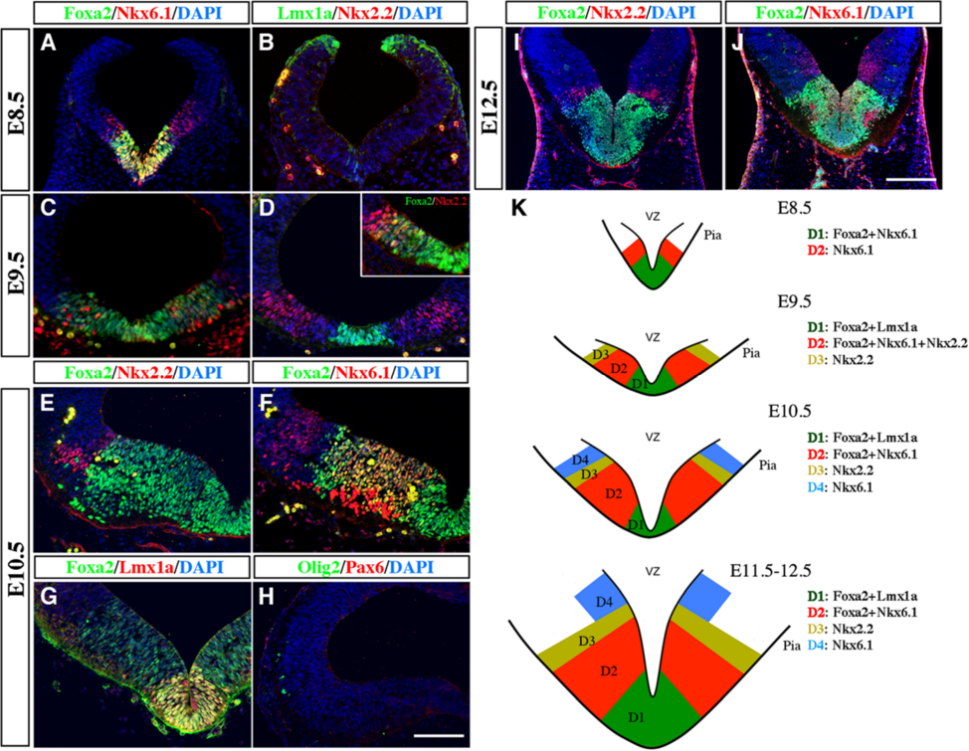
**Distinct progenitor domains in the developing ventral midbrain (vMB) defined by a combinatorial code of transcription factors.** (**A-D**) Double immunostaining for (**A,C**) Foxa2/Nkx6.1, (**B,D**) Lmx1a/Nkx2.2 and (inset) Foxa2/Nkx2.2 in vMB at embryonic day (E)8.5 (nine somites) and E9.5. (**A**) At E8.5, Foxa2 and Foxa2/Nkx6.1 defines domain D1 and D2. (**B**) Note that Lmx1a and Nkx2.2 are not expressed at E8.5. (**C,D**) At E9.5, Lmx1a/Foxa2, Foxa2/Nkx6.1, and Nkx2.2 define domains D1 to D3, respectively. (**E-J**) Confocal images show the expression pattern of (**E,I**) Foxa2/Nkx2.2, (**F,J**) Foxa2/Nkx6.1, (**G**) Foxa2/Lmx1a and (**H**) Olig2/Pax6 in vMB at E10.5 and E12.5. At both stages, Lmx1a/Foxa2, Foxa2/Nkx6.1, Nkx2.2-only and Nkx6.1-only define domains D1 to D4, respectively. Unlike in the ventral spinal cord, Olig2 and Pax6 were not detected in vMB at E10.5 Scale bars: (**H**) 50 μm, applied to **A-H**; (**J**) 100 μm, applied to I-J. (**K**) Schematic diagrams illustrating the D1 to D4 progenitor domains in vMB defined by a combinatorial code of transcription factors from E8.5 to E12.5.

In the ventral spinal cord, distinct progenitor domains have been identified based on different expression patterns of homeodomain transcription factors, such as Nkx2.2, Nkx6.1, and Olig2. These progenitor domains generate distinct classes of neurons in response to Shh signaling [24,25]. To determine if the developing vMB also contains different progenitor domains, we analyzed the temporal and spatial expression patterns of several transcriptional factors, including Lmx1a, Foxa2, Nkx6.1, and Nkx2.2, which have been implicated in neurogenesis in this region [17,26,27]. As described below, we found that a combinatorial set of transcription factor expression did indeed define distinct progenitor domains in vMB. These progenitor domains, termed ventral midbrain domain (D)1 to D4, followed a medial to lateral expansion as the embryos became more mature. Upon closure of neural tube at E8 to E8.5, the vMB contained two distinct domains, with the medial domain D1 expressing Foxa2 and Nkx6.1, whereas the immediately adjacent lateral domain D2 expressed Nkx6.1 only. At this stage, there was no detectable expression of Lmx1a or Nkx2.2 (Figure 1A,B,K). At E9.5, expression of Lmx1a emerged, and its coexpression with Foxa2 defined the newly formed medial domain D1, whereas the Foxa2 and Nkx6.1 co-expressing cells shifted laterally to become the D2 domain (Figure 1C,D,K). Immediately adjacent to the domain D2 was a small D3 domain that expressed Nkx2.2, although a small number of the Nkx2.2+ cells could also be detected within the D2 domain (Figure 1D and inset). After E9.5, the vMB showed tremendous expansion, and now contained four distinct domains defined by these transcription factors. From E10.5 to E12.5, the medial-most domain D1 expressed Lmx1a+Foxa2+ cells, and the adjacent D2 domain expressed Foxa2+Nkx6.1+ cells. The more lateral D3 domain expressed Nkx2.2, whereas a small population of cells positive for Nkx6.1 only defined the D4 domain (Figure 1E-K). Interestingly, unlike in spinal cord, expression of Olig2 and Pax6 could not be detected in midbrain at E10.5, suggesting that progenitor domains in vMB contained distinctly different transcriptional profiles from those in spinal cord.

Taken together, these results highlighted the dynamic expansion of the progenitor domains from E8.5 to E12.5 in the developing vMB. Furthermore, they provided an important framework to investigate potential effects of exogenous and intrinsic mechanisms that might affect the generation of DA neurons and other neuron subtypes at these critical developmental stages.

### Dynamic expression of Shh and Shh downstream effectors in the developing ventral midbrain

It has been well established that temporal adaption to gradient Shh signaling specifies the formation of different progenitor domains in the ventral spinal cord, and thereby controls the generation of different classes of neurons [1,5,25,28]. To understand the roles of Shh signaling in controlling the formation of progenitor domains and generation of different classes of neurons in the developing vMB, we characterized the spatial and temporal expression patterns of Shh and Shh downstream signaling effectors, including Smoothened, Patched, Gli1, Gli2, and Gli3.

**Figure 2:**
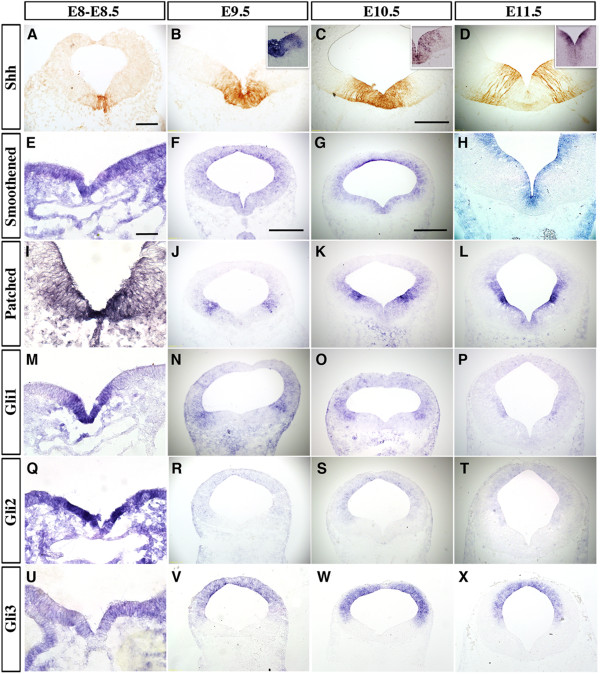
**Dynamic expression patterns of Shh signaling components in the developing ventral midbrain (vMB).** (**A**) Immunohistochemical staining and *in situ* hybridization (insets) revealed Shh expression in the midline of vMB at embryonic day (E)8.5 (10 somites). (**B-C** and insets) Shh expression in vMB extended laterally from E9.5 toE10.5, then (**D**, inset) became restricted laterally at E11.5. (**E-H**) *In situ* hybridization for *Smoothened* from E8 to E11.5 show that Smoothened was diffusely expressed both dorsally and ventrally in midbrain and its expression became progressively restricted to the ventricular zone in vMB at E10.5 to E11.5. (**F, G, H**) Dashed lines outline the pia side of neurotube. (**I-X**) *In situ* hybridization for (**I-L**) *Patched*, (**M-P**) *Gli1*, (**Q-T**) *Gli2*, and (**U-X**) *Gli3* from E8 to E11.5. All were expressed medially at E8 to E8.5 (7–10 somites), and then shifted laterally from E9.5 onwards. Scale bars: (**A**), 50 μm, applied to **A, B**; (**C**) 200 μm, applied to **C, D, H**; (**E**) 25 μm, applied to **E, I M, Q, U**; (**F**) 200 μm, applied to **F, J, N, R, V**; and (**G**) 500 μm, applied to **G, K, O, S, W, L, P, T, X**.

Consistent with previous studies [13,15,29], we found that Shh expression, detected by immunohistochemistry and *in situ* hybridization, showed robust and dynamic expression in vMB from E8 to E12.5. At E8 to E8.5, Shh proteins and *Shh* mRNA were detected mainly in the most medial region in vMB, and this expression domain expanded laterally from E9.5 to E10.5. Interestingly, from E11.5 to E12.5, *Shh* mRNA expression diminished in the most medial vMB D1 domain, and became more restricted to the ventricular zone (VZ) of the lateral D2 domain in vMB (Figure 2A-D, and insets in Figure 2B-D). Similar to our previous results [29], Shh proteins were detected in radial glial processes extending from the ventricular zone to the marginal zone at E11.5 (Figure 2D).

Unlike the dynamic changes of Shh expression in vMB, we found that *Smoothened*, one of the receptors for Shh, showed a rather diffuse expression pattern that covered both ventral and dorsal parts of the developing midbrain from E8.5 to E10.5. From E10.5 onward, *Smoothened* expression became more restricted to the ventricular zone within the vMB (Figure 2E-H). In addition to examining *Smoothened*, we also examined the expression patterns of several Shh signaling effectors, including *Patched*, *Gli1*, *Gli2*, and *Gli3*[26]. We found that Patched and Gli1 were both transiently expressed in the ventral medial region at E8 to E8.5. From E9.5 onward, the expression of Patched and Gli1 shifted laterally, and became more prominent in the ventricular zone of vMB D3 and D4 domains (Figure 2I-P). The expression pattern of Gli2 resembled those of Patched and Gli1, with a very robust level in the D1 and D2 domains at E8 to E8.5 (Figure 2Q), and shifting laterally and dorsally from E9.5 onwards (Figure 2R-T). Finally, Gli3, the major repressor of Shh signaling, showed low and diffuse expression in the vMB at E8 to E8.5 (Figure 2U), but its expression became restricted to the dorsal part of midbrain after E9.5 (Figure 2V-X).

**Figure 3:**
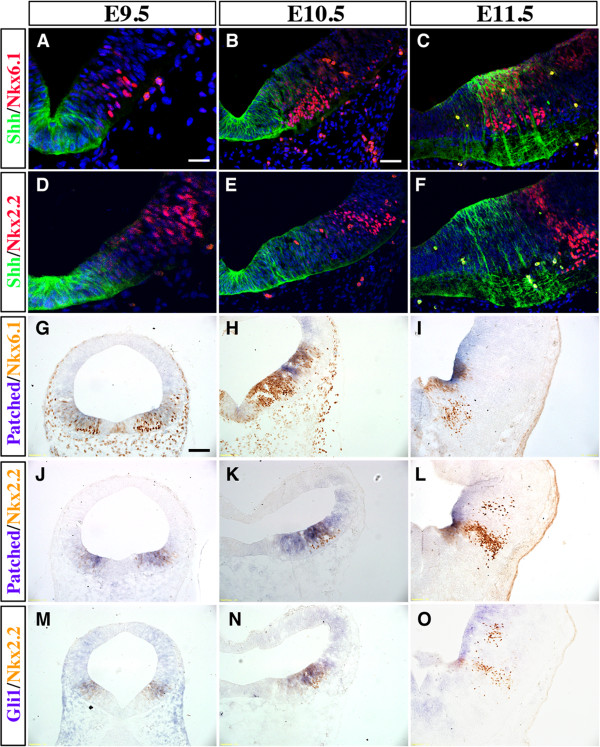
**Spatial distribution of Sonic hedgehog (Shh) signaling effectors in ventral midbrain progenitor domains.** (**A-C**) Double immunofluorescence staining of Shh and Nkx6.1 reveal that Shh proteins were expressed mainly in (**A**) the D1 region at embryonic day (E)9.5,(**B**) D1 and D2 regions at E10.5, and (**C**) D2 region at E11.5. (**D-F**) Double immunofluorescence staining of Shh and Nkx2.2 show partial colocalization from E9.5 to E10.5, but distinct separation at E11.5. (**G-O**) Combined *in situ* hybridization of *Patched* and *Gli1* with immunohistochemistry for Nkx6.1 and Nkx2.2 show that Patched and Gli1 mRNA were expressed in D2 to D4 domains, especially enriched in the D3 region. Scale bars: (**A**) 25 μm, applied to **A, D**; (**B**) 50 μm, applied to **B-C** and **E-F**; (**G**) 100 μm, applied to **G-O**.

Because the expression patterns of *Shh*, *Patched* and *Gli1* showed a medial to lateral expansion from E8.5 to E12.5 (Figure 1; Figure 2), we investigated if they might overlap with the vMB progenitor domains. Consistent with this idea, we found that Shh proteins were close to or partially overlapping with the Nkx6.1+Nkx2.2+ D2 domain at E9.5 and E10.5 (Figure 3A,B,D,E). By E11.5, the Shh proteins showed extensive overlapping with the Nkx6.1+ D2, domain and were immediately adjacent to the Nkx2.2+ D3 Domain (Figure 3C,F). Using combined *in situ* hybridization and immunohistochemistry, we found that the expression of *Patched* mRNA covered the D2 and D3 progenitor domains at E9.5 and E10.5 (Figure 3G,H,J,K), but became more restricted to the ventricular zone after E11.5 (Figure 3I,L). Similar to Patched expression, *Gli1* mRNA also showed extensive overlap with the D2 and D3 domains at E9.5 and E10.5 (Figure 3M,N), but at E11.5, the *Gli1* mRNA continued its expression in the D3 and D4 domains (Figure 3O).

Based on the dynamic, yet significant overlapping, expression of Shh signaling effectors in the vMB progenitor domains, these results suggest that Shh signaling might affect the temporal and spatial development of medial progenitors before E10.5. After E11.5, the lateral domains were the major regions receiving Shh signals. These results are reminiscent of the medial to lateral shift of progenitor domains in ventral spinal cord [4,5], and suggest that the effects of Shh on the progenitors and neurons arising from the medial domains could be transient, whereas the effects on progenitors and neurons arising from lateral domains could last longer.

### Removal of Smoothened in ventral midbrain leads to a transient reduction in ventral progenitors

**Figure 4:**
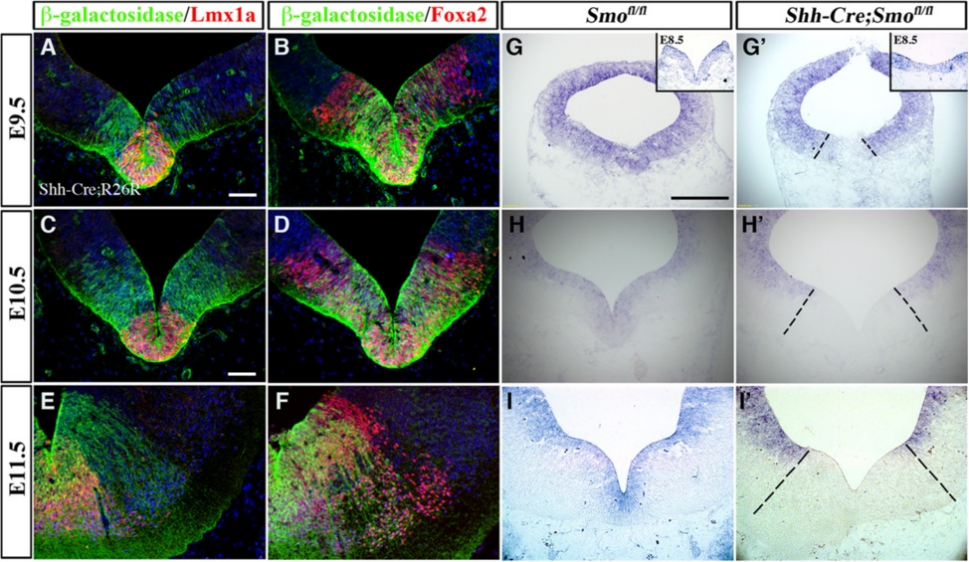
**Region-specific removal of Smoothened in the ventral midbrain of *Shh-Cre;Smo***^***fl/fl***^**mutants.** (**A-F**) Using the R26R reporter line, we found that *Shh-Cre* drives recombination in D1 domain and the majority of D2 domain from embryonic day (E)9.5 to E11.5. (**G-I**’) *In situ* hybridization of *Smoothened* indicates complete removal of *Smoothened* in *Shh-Cre;Smo*^*fl/fl*^ mice from E9.5 to E11.5, and (**G-G**’ and insets) incomplete removal at E8.5 (eight somites). Scale bars: (**A**) 25 μm, applied to **A-B**; (**C**) 50 μm, applied to **C-F**; (**G**) 200 μm, applied to **G** and **I**’.

To examine the roles of Shh signaling in the development of ventral midbrain, we generated conditional knockout mice in which the Shh receptor, Smoothened, was removed in vMB using *Shh-Cre* (named *Shh-Cre;Smo*^*fl/fl*^). Consistent with the previously reported activity of *Shh-Cre* in vMB [21], we found that the *Shh-Cre* recombination pattern completely covered the Lmx1a+ D1 domain and the majority of the Foxa2+ D2 domain from E9.5 to E11.5 (Figure 4A-F). Using *in situ* hybridization, we confirmed complete removal of *Smoothened* mRNA in vMB from E9.5 to E11.5 (Figure 4G-I’). The reduction in levels of *Smoothened* mRNA in *Shh-Cre;Smo*^*fl/fl*^ mutants began as early as E8.5 (eight-somite stage) (Figure 4G-G’, insets); this reduction was partial.

**Figure 5:**
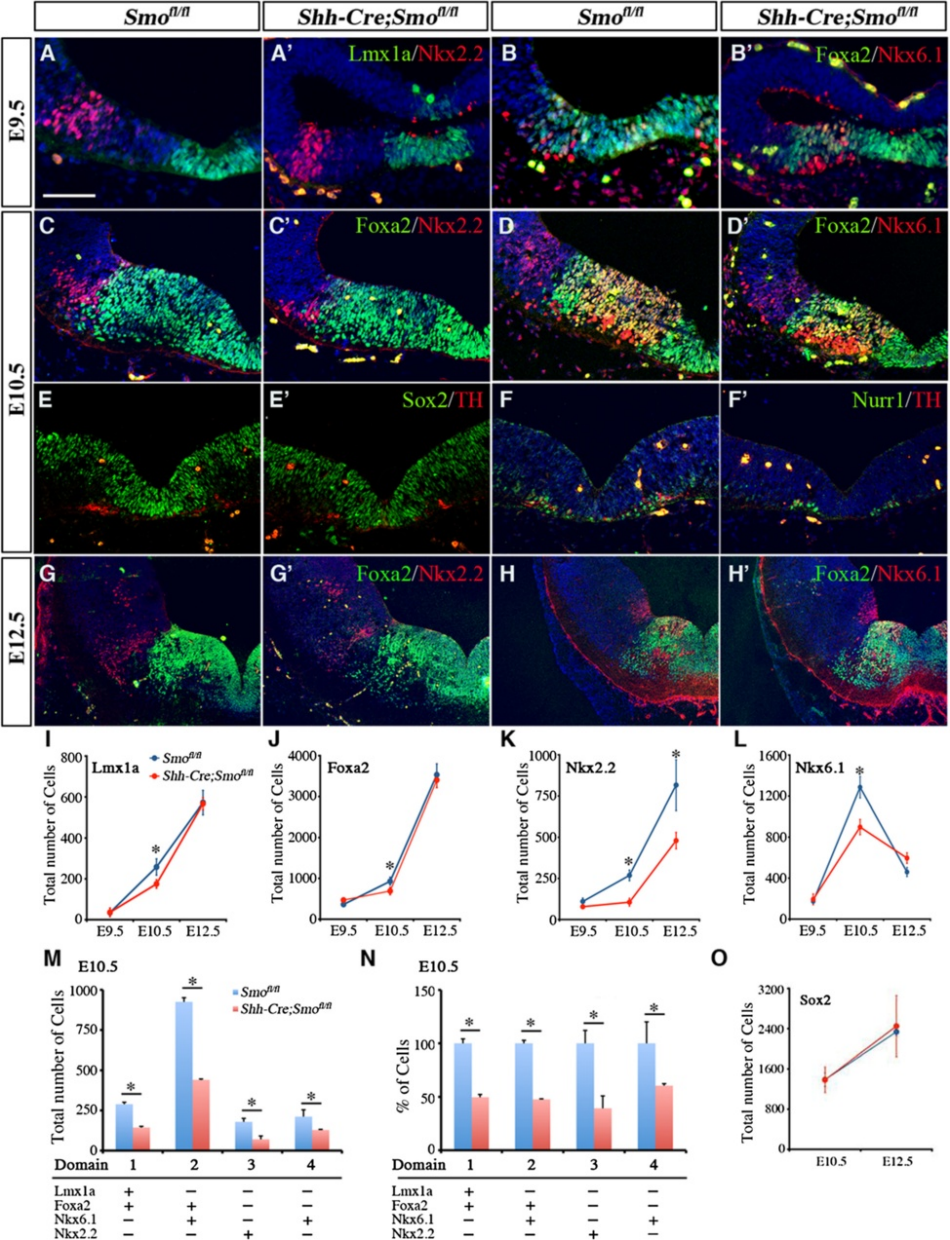
**Transient reduction of dopaminergic (DA) progenitors in *Shh-Cre;Smo***^***fl/fl***^**mutants.** (**A-B**’) At embryonic day (E)9.5, *Shh-Cre;Smo*^*fl/fl*^ mutants showed no detectable reduction in progenitors expressing (**A**’) Lmx1a/Nkx2.2, or (**B**’) Foxa2/Nkx6.1. (**C-F**’) By contrast, *Shh-Cre;Smo*^*fl/fl*^ mutants showed a significant reduction in (**C-C**’, **D-D**’) Foxa2+, (**C-C**’) Nkx2.2+, (**D-D**’) Nkx6.1+, and (**F-F**’) Nurr1+ progenitors at E10.5, whereas (**E-E**’) Sox2+ progenitors were unchanged. (**E-F**’) Note that the DA neurons were also reduced at E10.5. (**G’,H**’) Foxa2+ and (**H**’) Nkx6.1+ progenitors from E12.5 *Shh-Cre;Smo*^*fl/fl*^ mutants showed no change compared with (**G**,**H**) controls, whereas (**G**’) the reduction in Nkx2.2+ progenitors persisted at E12.5. Scale bars: (**A**) 50 μm (applied to **A** to **H**’). (**I-L**) Quantification of total number of (**I**) Lmx1a+, (**J**) Foxa2+, (**K**) Nkx2.2+ and (**L**) Nkx6.1+ progenitors confirmed their transient reduction at E10.5. Student’s *t* test, n = 3 or 4. (**M-N**) Quantification of number of cells in each progenitor domains at E10.5 showed a consistent reduction from D1 to D4 domains at E10.5.

To analyze the effects of ablating Smoothened, we quantified the numbers of progenitors in each vMB domain (D1 to D4). Surprisingly, despite the robust expression of Shh signaling effectors in vMB, we found no detectable reduction in the number of progenitor cells at E8.5 to E9.5 (Figure 5A-B’, and data not shown). Beginning at E10.5, there was a consistent decrease in the number of Lmx1a+, Foxa2+, Nurr1+, Nkx6.1+ and Nkx2.2+ progenitors in D1 to D4 domains in *Shh-Cre*;*Smo*^*fl/fl*^ embryos (Figure 5C-D’,I-N). Despite this reduction, the spatial arrangement of D1 to D4 domains was not altered (Figure 5C-D’). Furthermore, we did not detect any changes in the Sox2+ progenitors (Figure 5E-E’). The changes were not due to changes in cell death or proliferation, because there were no detectable changes in caspase 3 staining or 2-hour BrdU incorporation (data not shown). The effects of Smoothened loss of function on most progenitors appeared to be transient, thus by E12.5, there were no detectable differences in the total number of progenitors, including those for Lmx1a, Foxa,2 and Nkx6.1 (Figure 5G-L). Only the Nkx2.2+ progenitors in the D3 domain continued to show a significant reduction at E12.5 (Figure 5G-G’,H,K). Together, these data support the idea that the loss of Smoothened had a transient effect on the expansion of most progenitors in vMB at E10.5, except for the Nkx2.2+ progenitors, which showed persistent reduction at E12.5.

### Loss of Smoothened in ventral midbrain affects the generation of neurons in red nucleus, oculomotor nucleus, and raphe nuclei, but not dopaminergic neurons

**Figure 6:**
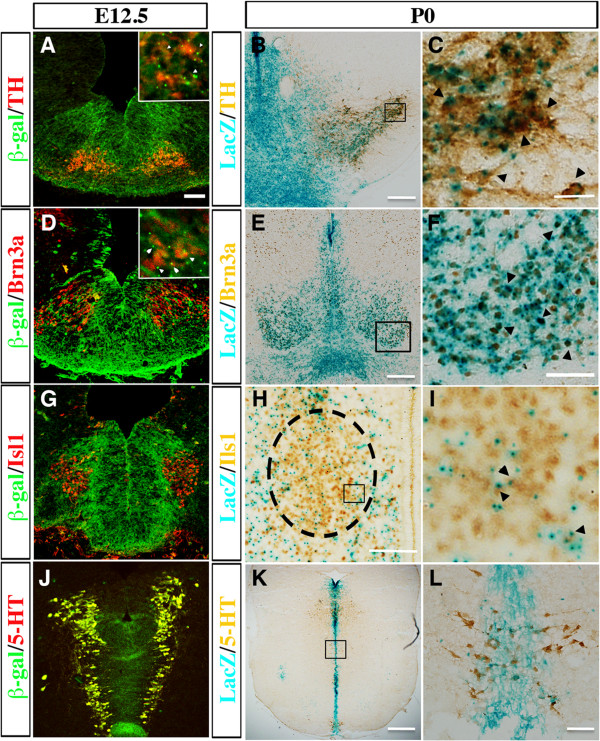
**Fate mapping of dopaminergic neurons, red nucleus neurons, oculomotor neurons, and serotonergic neurons from Sonic hedgehog (Shh)-expressing cells.** Using the *Shh-Cre;R26R* reporter line, we found that (**A**) tyrosine hydroxylase (TH)+ DA neurons, (**D**) Brn3a+ neurons in the red nucleus, (**G**) Isl1+ oculomoter neurons (**H**) and 5-hydroxytryptamine (5-HT)+ serotonergic neurons showed extensive coexpression of β-galactosidase at embryonic day (E)12.5. (**A** and **D** insets) Arrowheads show the colocalization of (**A** inset) TH (red) and (**D** inset) Brn3a (red) with β-galactosidase (green) at E12.5. At post-natal day (P)0, (**B**) TH+, (**E**) Brn3a+, (**H**) Isl1+ and (**K**) 5-HT+ neurons expressed within the LacZ-expressed regions (blue). (**C**,**F**,**I**,**L**) Higher magnifications of the boxed regions in (**B**,**E**,**H**,**K**), respectively. Black arrowheads indicate the colocalized cells. Scale bars: (**A**) 50 μm, applied to **A, D, G, E**); (**B, F, H**) 100 μm; (**C**) 50 μm; (**E**) 200 μm.

Given the progressively restricted expression of Shh effectors to the lateral domains in vMB, we investigated if the effects of Smoothened loss of function on the progenitors at E10.5 and E12.5 might affect distinct classes of neurons arising from different vMB domains (Figure 2, Figure 3, Figure 5). The embryonic vMB gives rise to four major subtypes of neurons: 1) DA neurons, 2) neurons in the red nucleus, 3) oculomotor neurons, and 4) serotonergic neurons. It is known that the DA neurons are generated from the Foxa2+Lmx1a+ D1 domain from E10.5 to E12.5, the oculomotor neurons from the Nkx6.1+Foxa2+ D2 domain, and the red nucleus neurons from the Nkx6.1+Foxa2+ D2 domain, while the serotonergic neurons partially arise from the Nkx2.2+ progenitors in the caudal vMB [17,26,27,30,31]. To confirm these results, we used genetic-fate mapping to investigate whether these four groups of neurons derived from Shh-expressing cells. Using anti-β-Gal antibody or colorimetric LacZ expression in *Shh-Cre;R26R/+* mice, we could detect β-GAL coexpressed with all the TH+, Brn3a+, Islet1+ and 5-HT+ neurons at E12.5 in the midbrain. By post-natal day (P)0, LacZ expression could still be detected in most TH+ and Brn3a+ neurons, and in a significant number of Isl1+ and 5-HT+ neurons (Figure 6A-L). These results indicated that DA neurons (TH+), red nucleus neurons (Brn3a+), oculomotor neurons (Isl1+) and serotonergic neurons (5-HT+) were completely or partially derived from Shh-expressing progenitors, and *Shh-Cre* could be an effective tool to target these groups of neurons.

**Figure 7:**
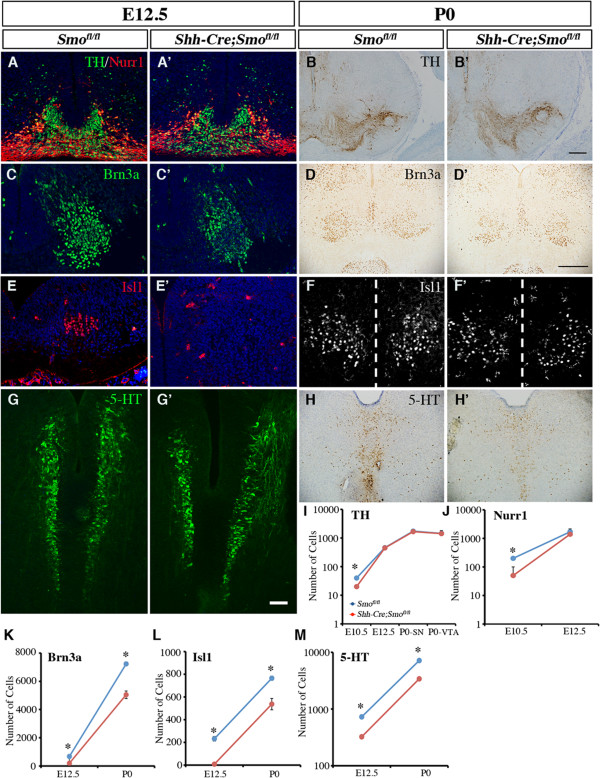
**Persistent loss of neurons in the red nucleus, oculomotor nucleus and the serotonergic neurons, but not dopaminergic neurons, in *Shh-Cre;Smo***^***fl/fl***^**mutants at embryonic day (E)12.5 to post-natal day (P)0.** (**A-B**’) Nurr1 and tyrosine hydroxylase (TH) staining from E12.5 and P0 *Shh-Cre;Smo*^*fl/fl*^ mutants showed no change compared with controls. (**C-D**’) Brn3a, (**E-F**’) Islet1 and (**G-H’**) 5-Hydroxytryptamine (5-HT) revealed selective reduction of neurons in the red nucleus, neurons in the oculomotor nucleus, and serotonergic neurons in the raphe nuclei at E12.5 and P0 after removal of Smoothened. (**F-F’**) Dashed line indicates the midline. Scale bars: (**G’**) 50 μm, applied to **A-A**’, **C-C**’, **E-E**’ and **G-G**’). (**I-J**) Quantification of TH+ and Nurr1+ cells at E10.5, E12.5 and P0 confirmed the transient reduction of committed DA progenitors and DA neurons in *Shh-Cre;Smo*^*fl/fl*^ mutants. (**K-M**) Quantification of the reduction of neurons in the red nucleus, oculomotor nucleus, and the raphe nuclei (serotonergic neurons) at E12.5 and P0 in *Shh-Cre;Smo*^*fl/fl*^ mutants. Student’s *t* test, n = 3 or 4.

Using cell type-specific markers, we found that the number of TH+ DA neurons and Nurr1+ DA intermediate progenitors, which were derived from Lmx1a+Foxa2+ progenitors in the D1 domain, were transiently reduced in the *Shh-Cre;Smo*^*fl/fl*^ mutants at E10.5 (Figure 5F-F’). Interestingly, by E12.5, there was no detectable reduction in the population of either cell type (Figure 7A-B’, I-J). By contrast, the numbers of Brn3a+ red nucleus neurons and Isl1+ oculomotor neurons, which both arose from the Nkx6.1+/Foxa2+ progenitors in the D2 domain, were significantly decreased at E12.5, and this decrease persisted at P0. Furthermore, we also detected a persistent reduction of serotonergic (5-HT+) neurons at E12.5 and P0 (Figure 7C-H’,K-M). Taken together, these data showed that the loss of Smoothened in the vMB had a transient and modest effect on the generation of DA neurons and DA progenitors at E10.5, but a more persistent effect on the generation of Brn3a+, Isl1+, and 5-HT+ neurons from the more lateral D2 and D3 domains.

### Constitutive activation of smoothened transiently expand progenitors in ventral midbrain

**Figure 8:**
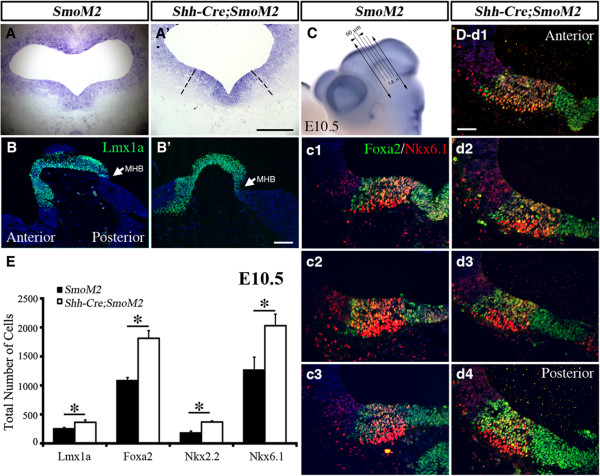
**Constitutive activation of Sonic hedgehog (Shh) signaling in *Shh-Cre;SmoM2 *mutants leads to transient expansion of progenitors in ventral midbrain.** (**A-A**’) *In situ* hybridization of Smoothened indicated overexpression of Smoothened in *Shh-Cre;SmoM2* mutants at embryonic day (E)10.5. (**B-B**’) Immunofluorescence staining of Lmx1a on sagittal sections revealed the anterior extension of Lmx1a domains from vMB in *Shh-Cre;SmoM2* mutants (**B**’) at E10.5. Arrow indicates the midbrain/hindbrain boundary (MHB). (**C**) Illustration of how a series of coronal sections were generated in the ventral midbrain (vMB) at E10.5. Whole-mount staining of Wnt1 outlines the vMB region. Coronal sections were generated by cutting vMB at 60 μm intervals. (**c1-d4**) Foxa2/Nkx6.1 staining: (**c1-c3**) in control *SmoM2* mice, there were three sections with vMB floor plate feature, whereas (**d1-d4**) there were four sections with this feature in *Shh-Cre;SmoM2* mutants. Scale bars: (**A**) 200 μm, (**B’**) 100 μm, applied to **B** and **B**’; (**C**’) 100 μm, applied to **C** and **C**’; (d’-1) 100 μm, applied to c1 to d’4. (**E**) Quantification of total number of Lmx1a, Foxa2, Nkx2.2 and Nkx6.1 cells show the increase in progenitor cells at E10.5. Student’s *t* test, n = 3 or 4.

To further examine the role of Shh signaling in controlling vMB development, we generated mice that expressed constitutively active Smoothened receptor under the control of *Shh-Cre* (named *Shh-Cre;SmoM2)*[32]. Owing to the constitutive activation of Smoothened in the Shh expression domains, essentially all the *Shh-Cre;SmoM2* showed limb malformations, with abnormal growth of cartilage in the patterning center of the developing limbs (data not shown). As expected, the expression of an extra copy of Smoothened transcript led to more intense *Smoothened* mRNA signals detected by *in situ* hybridization (Figure 8A-A’).

Because removal of Smoothened led to a transient reduction in vMB progenitors, we investigated if constitutive activation of Smoothened might have the opposite effect. As anticipated, we detected an expansion of vMB in *Shh-Cre;SmoM2* mice. Interestingly, several lines of evidence indicated that the expansion of vMB in *Shh-Cre;SmoM2* mice occurred along the anterior-posterior (A-P) axis. First, by sectioning E10.5 vMB in the coronal plane at 60 μm intervals, we detected more sections that contained the progenitor domains in vMB of *Shh-Cre;SmoM2* mice compared with *SmoM2* controls (Figure 8C-D,c1-3,d1-4). As a consequence, the total number of progenitors in vMB labeled by Lmx1a, Foxa2, Nkx2.1, and Nkx6.1 showed significant increases at E10.5 (Figure 8E). Second, when examined on sagittal planes, the Lmx1a expression domain was seen to extend anteriorly in *Shh-Cre;SmoM2* mice, whereas the posterior boundary of he Lmx1a+ domain ended in the same MHB in both control and *Shh-Cre;SmoM2* mutants (Figure 8B-B’). Such expansion of the vMB was not detected at E9.5 or E12.5 (data not shown). Together, these results were consistent with the Smoothened loss-of-function data (Figure 5, Figure 7), and further confirmed that the effect of Smoothened signaling had transient effects on the development of progenitors in vMB.

### Smoothened antagonizes Wnt signaling in dopaminergic neuron development in ventral midbrain

**Figure 9:**
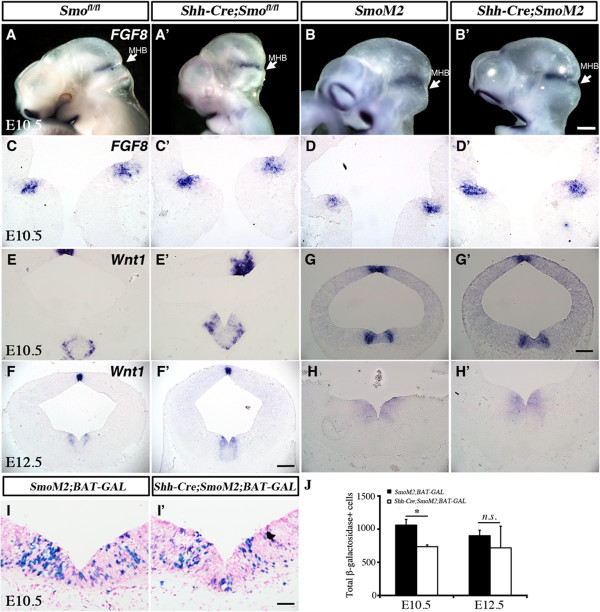
**Loss and gain of function in Smoothened affects *Wnt1*, but not fibroblast growth factor (*FGF*)8, expression, in ventral midbrain (vMB).** (**A-D**’) *In situ* hybridization showed no detectable change in *FGF8* expression in MHB in either (**A**’**,C**’) *Shh-Cre;Smo*^*fl/fl*^ or (**B**’**,D**’) *Shh-Cre;SmoM2* mutants compared with (**A**,**C** and **B**,**D**) controls. (**E-F**’) *Wnt1* mRNA was increased in vMB of *Shh-Cre;Smo*^*fl/fl*^ at both embryonic day (E)10.5 and E12.5. (**G**) *Wnt1* mRNA was slightly decreased in vMB of *Shh-Cre;SmoM2* at E10.5, but (**H**’) resumed at E12.5. (**I-I**’) LacZ staining from *BAT-GAL* reporter indicate that (**I**’) the number of Wnt-responsive cells was decreased in vMB of *Shh-Cre;SmoM2;BAT-GAL* mutants at E10.5, but returned to the same level as controls at E12.5 (data not shown). (**I**) Quantification confirms the decrease of Wnt-responsive cells in vMB of *Shh-Cre;SmoM2;BAT-GAL* mutants at E10.5, which returns to the same level as controls at E12.5. Student’s *t*-test, n = 3 or 4. Scale bars: (**B**’) 500 μm, applied to **A** to **B**’; (**G**’), 100 μm, applied to **C-E**’ and **G-H**’; (**F’**) 200 μm, applied to **F-F**’; (**H**) 500 μm, applied to **H-H**’; (**I**’), 100 μm, applied to **I-I**’.

Given the fact that there was no detectable difference in DA lineage cells between either *Smo*^*fl/fl*^ and *Shh-Cre*;*Smo*^*fl/fl*^ or *SmoM2* and *Shh-Cre;SmoM2* at E12.5, the results (Figure 5, Figure 6, Figure 7, Figure 8) suggested that other signaling pathway(s) might have stage-dependent effects in regulating DA neuron development after E12.5. It has been shown that in addition to Shh and Wnt signaling, FGF8 is required for the patterning of MHB, induction of midbrain DA neurons, and regulation of DA progenitor domains [12,21,33]. By examining *FGF8* mRNA on whole-mount animal and sections from E9.5 to E12.5, we found that no difference in *FGF8* expression could be detected in MHB, either in *Shh-Cre*;*Smo*^*fl/fl*^ or *Shh-Cre;SmoM2* mutants (Figure 9A-D’ and data not shown). These results were distinctly different from those reported in *En1-Cre;Smo*^*fl/fl*^ mutants, in which a marked reduction in the expression of FGF8 in midbrain-hindbrain region caused a profound patterning defect [8,17].

Previously, we reported that stabilizing the canonical Wnt signaling antagonized Shh expression in vMB to control the temporal development of DA neurons [29]. To examine the effect of Shh signaling on Wnt1 expression, we performed *in situ* hybridization to examine the expression of *Wnt1* mRNA in both *Shh-Cre*;*Smo*^*fl/fl*^ and *Shh-Cre;SmoM2* mutants. Consistent with our prediction, at both E10.5 and E12.5, *Wnt1* mRNA levels were increased in the vMB of *Shh-Cre*;*Smo*^*fl/fl*^ compared with control (Figure 9E-F’). By contrast, in the *Shh-Cre;SmoM2* mutant, *Wnt1* mRNA level was modestly downregulated at E10.5, but returned to the control levels at E12.5 (Figure 9G-H’).

To further investigate the effects of Smoothened on Wnt signaling, we generated *SmoM2;BAT-GAL* and *Shh-Cre;SmoM2;BAT-GAL* mice, in which the Wnt signaling reporter, *BAT-GAL*, could be used as a surrogate for canonical Wnt activity [20,34]. Consistent with the *Wnt1* mRNA changes in *Shh-Cre;SmoM2* mutants, quantification of the number of β-Gal+ cells showed a significant reduction at E10.5 in *Shh-Cre;SmoM2;BAT-GAL*, but returned to the control level at E12.5 (Figure 9I-J). Collectively, these data suggest that there is a mutual antagonism effect between Shh and Wnt signaling in vMB. Perturbations in the Shh signaling mechanism triggered a transient, compensatory activation of Wnt signaling on vMB at E10.5.

## Discussion

**Figure 10:**
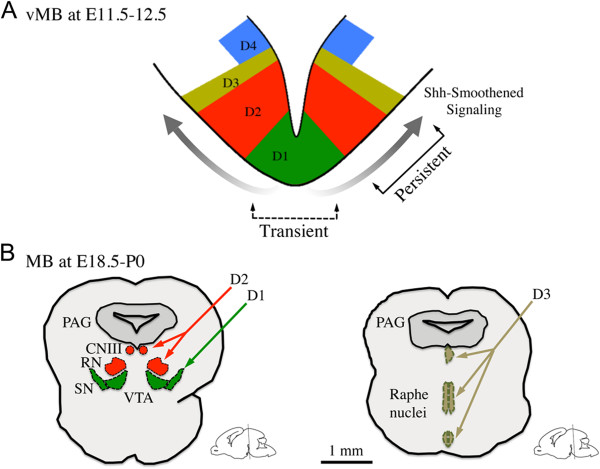
**A working model for the influences of Sonic hedgehog (Shh)-Smoothened signaling in neuron development from ventral midbrain.** (**A**) Shh-Smoothened signaling exerted spatial and temporal influences on the progenitors in vMB at embryonic day (E)11.5 to 12.5. (**B**) Loss of Shh-Smoothened signaling resulted in more pronounced and persistent effect of differentiated neurons in the oculomotor nucleus (CNIII), red nucleus (RN), and the raphe nuclei. PAG, periaqueductal grey; SN, substantia nigra; VTA, ventral tegmental area.

In this study, we characterized the temporal and spatial expression patterns of neural progenitors in the vMB during early embryogenesis, and determined how Shh-Smoothened signaling influences the development of these progenitors. Removal of Smoothened in vMB transiently reduced the Lmx1a+ and Foxa2+ DA progenitors at E10.5, but did not affect the subsequent DA neuron development after E12.5. Instead, loss of Smoothened led to persistent deficits in the Nkx2.2+ progenitors in the vMB at E10.5 to E12.5, and to the development of neurons from these progenitors, including the red nucleus neurons, oculomotor neurons, and serotonergic neurons. Conversely, expression of a constitutively active Smoothened in vMB resulted in the expansion of DA progenitors at the same stage, suggesting that the effects of Shh-Smoothened in the vMB progenitors are stage-dependent. Consistent with previous studies, we found that loss of Smoothened led to activation of Wnt1 signaling during the early development of vMB, supporting the idea of an antagonistic relationship between Shh-Smoothened and Wnt-β-catenin signaling in vMB [21,29,35]. Together, our data support the model that Shh-Smoothened controls vMB neuronal development in a temporal and spatial manner. At the early stage of vMB development, Shh-Smoothened signaling is transiently required for DA neuron development from the medial D1 domain (Figure 10A). As development progresses to the late embryonic and perinatal stages (E18.5 to P0), Shh-Smoothened signaling exerts a more pronounced and persistent effect on the more lateral D2 and D3 domain-derived neurons in the oculomotor nucleus (CNIII), the red nucleus (RN), and the raphe nuclei (Figure 10B).

### Dynamic progression of progenitor domains in early ventral midbrain development

Several lines of evidence indicate that the ventral region of the developing neural tube contain progenitors that can be divided into distinct domains based on the expression of cell type-specific transcriptional factors, which are required for the development of different groups of neurons in the ventral neural tube [1,4,5]. Although previous studies attempted to define the vMB progenitor domains based on the expression of transcriptional factors [36], their results do not provide the temporal resolution of these progenitor domains in the developing vMB at the stages when patterning, expansion, and differentiation of these progenitors are active. By contrast, our results show that a combinatorial code of cell type-specific transcription factors defines discrete progenitor domains in vMB that are distinctly different from the ventral progenitors in spinal cord. First, the progenitor domains in vMB, marked by Lmx1a, Foxa2, Nkx2.1, and Nkx6.1, are identified along the midline at E8 to E8.5 (D1 and D2 domains), and subsequently expand to the lateral domains from E9.5 to E12.5 (D3 and D4 domains) (Figure 1, Figure 3). Although such medial to lateral expansion in vMB is similar to the ventral to dorsal expansion in the spinal cord, the Foxa2+ progenitor domain undergoes a tremendous expansion in vMB as neurogenesis progresses, compared with its progressively more restricted pattern in the most ventral region of the spinal cord. Second, unlike the spinal cord, the Foxa2+ progenitors in vMB show extensive coexpression with Nkx6.1 and transient coexpression with Nkx2.2. Finally, our results showed no detectable Olig2 expression in vMB at E10.5, whereas Olig2 was expressed in the motor neuron progenitor (pMN) domain in spinal cord (Figure 1). Furthermore, the Pax6+ progenitors, which could be detected from pMN to p0 domains in the spinal cord, were distinctly absent in the vMB at E10.5. Together, our data clearly delineate the dynamic expansion of the vMB progenitor domains, which show important differences from those in the spinal cord.

### Sonic hedgehog-Smoothened signaling and neuronal development in ventral midbrain

Several studies have identified the Shh-expressing domain in vMB as an enriched source that gives rise to many neurons in the adult midbrain, including the DA neurons and neurons in the red nucleus [13-15]. Indeed, our results confirm and extend these findings by showing that the Shh signaling effectors are expressed in the medial D1 and D2 domains at E8 to E8.5. Interestingly, the Shh receptor *Smoothened* continued to show broad expression in vMB from E9.5 to 10.5, but became more restricted to the ventricular zone, especially in the neurogenic niche for DA progenitors, at E12.5 (Figure 2). By contrast, expression of *Patched* and *Gli1* shifted to the lateral D3 and D4 domains in vMB from E9.5 to 11.5, whereas expression of *Gli2* and *Gli3* was present primarily in the dorsal midbrain (Figure 2, Figure 3). Furthermore, our fate-mapping data show that the majority of the neurons in the red nucleus, oculomotor nucleus, and raphe nuclei are derived from progenitors that respond to Shh signaling (Figure 6). These results represent the first comprehensive view of the dynamic changes in the expression of Shh signaling effectors, and provide an important framework to understand how perturbation of Shh signaling might affect the development of neurons from the progenitors in vMB.

Intriguingly, despite the broad expression of Shh signaling effectors in vMB at E8 to E8.5, removal of Smoothened using *Shh-Cre* resulted in only a transient reduction in DA progenitors at E10.5. The delay in the onset of detectable loss of DA progenitors in the vMB of *Shh-Cre;Smo*^*fl/fl*^ mutants may be related to the slow turnover of Smoothened proteins after Cre recombination. Alternatively, the onset of *Shh-Cre*-mediated removal of Smoothened may not have completely removed Smoothened from the DA progenitors. Regardless of the exact mechanism, the DA progenitors in *Shh-Cre;Smo*^*fl/fl*^ mutants returned to the control level by E12.5. This modest and transient loss of the DA progenitors and DA neurons in *Shh-Cre;Smo*^*fl/fl*^ mutants is different from the severe DA neuron deficits seen in the *En1-Cre;Smo*^*fl/fl*^ or *En1-Cre;Shh*^*fl/fl*^ mutants [8,17], most likely due to the general patterning defects in dorsal and ventral midbrain caused by the *En1-Cre*. Consistent with this notion, *FGF8* expression, which is present in the MHB, is severely perturbed in both *En1-Cre;Smo*^*fl/fl*^ and *En1-Cre;Shh*^*fl/fl*^ mutants. By contrast, we did not observe any changes in *FGF8* expression either in *Shh-Cre*;*Smo*^*fl/fl*^ or *Shh-Cre;SmoM2* mutants (Figure 9A-D). *FGF8* has been shown to be required for the patterning of MHB, expansion of DA progenitors, and the induction of DA neurons [12,20,33]. Hence, perturbation to FGF8 expression in *En1-Cre;Smo*^*fl/fl*^ and *En1-Cre;Shh*^*fl/fl*^ mutants is likely to have a lasting effect on DA neurons owing to non-cell autonomous effects.

In contrast to the modest, transient phenotype in DA neurons, a pronounced and persistent deficit was noted in neurons derived from the more lateral D2 and D3 domains, including red nucleus neurons, oculomotor neurons, and serotonergic neurons (Figure 5, Figure 6, Figure 7). These results are consistent with the temporal and spatial requirements of Shh signaling in digit formation and ventral spinal cord development that have been shown previously by fate-mapping and genetic-ablation studies [5,37]. In addition, our results support the evolutionarily conserved function of Shh signaling on midbrain neuron development in chicks and mammals [38,39]. Perturbations to Shh-Smoothened signaling are likely to contribute to congenital defects involving midbrain neurons that are critical for extraocular movement, autonomic functions, and control of locomotion and respiratory rhythms [40-42].

### Antagonistic effects between Shh and Wnt signaling in dopaminergic neuron development

Both loss-of-function and gain-of-function analyses of β-catenin in vMB have shown that canonical Wnt signaling antagonizes Shh expression during the neurogenesis of DA neurons [29,35]. Such effects of Wnt and Shh have also been confirmed for the generation of DA neurons from stem cells [43]. Using *in situ* hybridization for *Wnt1* expression, we found increased *Wnt1* expression in the neurogenic niche for DA neurons in *Shh-Cre*;*Smo*^*fl/fl*^ mutants (Figure 9E-F’). Conversely, the Smoothened gain-of-function mutants *Shh-Cre;SmoM2* mutants exhibited reduced BAT-GAL reporter activity, indicating that the canonical Wnt activity is reduced in these mutants (Figure 9). Despite the increase in Wnt1 expression, however, *Shh-Cre*;*Smo*^*fl/fl*^ mutants showed a decrease in the DA progenitors at E10.5, suggesting that Shh-Smoothened activity, but not canonical Wnt signaling, has a more dominant effect in regulating the DA progenitor development in vMB at this stage (Figure 5). These results are consistent with our previous observations that stabilization of Wnt-β-catenin signaling using *Shh-Cre* expands DA progenitors only after E12.5, despite the fact that *Shh-Cre* recombination occurs as early as E9.5 [29].

## Conclusion

In conclusion, our study shows that region-specific removal of Smoothened in vMB has a surprisingly modest and transient effect in the development of DA progenitors and DA neurons. By contrast, loss of Smoothened has more severe and persistent effects on the neurons derived from lateral domains of the vMB. These results provide important insights to the previously unrecognized roles of Shh-Smoothened in the development of neurons that are critical to the control of extra-ocular movement, locomotion, and respiratory rhythms.

## Competing interests

The authors declare no competing financial interests.

## Authors’ contributions

MT performed and analyzed the majority of the experiments and helped design the experimental strategy. SXL and VT helped with some of the data. EJH oversaw the whole project and wrote the manuscript together with MT and SXL. All authors read and approved the final manuscript.
